# Pericardial Mesothelioma Presenting as Constrictive Pericarditis

**DOI:** 10.7759/cureus.24270

**Published:** 2022-04-19

**Authors:** Biraj Shrestha, Rishin Handa, Bidhya Poudel, Rittu Hingorani

**Affiliations:** 1 Internal Medicine, Reading Hospital Tower Health, Reading, USA; 2 Cardiology, Reading Hospital Tower Health, Reading, USA; 3 Internal Medicine, AMITA Health Saint Francis Hospital, Evanston, USA

**Keywords:** pericardiectomy, constrictive physiology, pericardial mass, constrictive pericarditis, pericardial mesothelioma

## Abstract

This case report presents a 60-year-old gentleman with a significant smoking history and possible asbestos exposure who was referred to the emergency department for atrial fibrillation with a rapid ventricular rate and symptoms of heart failure. Labs showed normal brain natriuretic peptide and troponin I. His echocardiography finding suggested constrictive pericarditis with an ejection fraction of 60%. A computed tomography scan was concerning for a pericardial mass. Left and right heart catheterization hinted more toward constrictive physiology; however, some findings were concerning for restrictive physiology. Hence, cardiac magnetic resonance imaging was done, which established the diagnosis of constrictive pericarditis. Pericardiectomy was planned with a maze procedure for atrial fibrillation. However, a malignant neoplasm was seen on a frozen biopsy. Hence, surgery was limited to partial pericardiectomy, as the patient had advanced infiltrative neoplasm that had resulted in constrictive pericarditis. The final pathology report confirmed the diagnosis of malignant pericardial mesothelioma mixed type. Malignancy is usually diagnosed in an advanced stage, like in our case, due to nonspecific initial presentation. A literature review suggests that there is a lack of established consensus on treatment. The response to therapy also seems to be poor and results only in palliation of symptoms, with a median survival of six months from diagnosis despite optimum medical management.

## Introduction

Malignant mesotheliomas are rare tumors in body cavities covered by mesothelium. The pleura is the most common site of its occurrence. However, it can also arise in other body sites like the peritoneum, pericardium, or tunica vaginalis of the testis [1]. Hillerdal et al. performed a large review of 4710 cases and reported that pericardial mesotheliomas accounted for only 0.7% of all mesotheliomas [2]. We report a rare case of primary pericardial mesothelioma presenting as constrictive pericarditis.

## Case presentation

A 60-year-old gentleman was referred to the emergency department after being found to be in atrial fibrillation with a rapid ventricular rate. He had a significant past medical history of chronic diastolic heart failure, permanent atrial fibrillation, and alcoholic cirrhosis. He is an active smoker with a 40-pack-year smoking history and a retired construction worker with reported asbestos exposure. On presentation, the patient complained of orthopnea, increased shortness of breath (New York Heart Association (NYHA) class 3), increased leg swelling, and palpitations over the last several weeks. He stated that he had gained 10 pounds unintentionally in the previous month. He denied chest pain, cough, nausea, dizziness, syncope, and fevers. His home medications included Eliquis, lasix, spironolactone, and metoprolol, with which he reported compliance. He denied any illicit drug use and had quit drinking six months ago. Of note, he reported having undergone a pericardial window for pericardial effusion a year ago at an outside facility.

**Figure 1:**
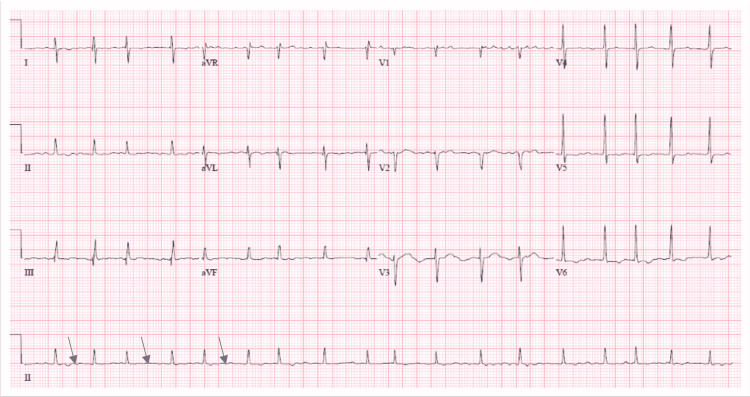
Electrocardiogram with blue arrows showing the fibrillary wave (see in atrial fibrillation) with a rapid ventricular rate of 108 beats per minute, with no significant ST-T wave changes

On examination, he was alert and oriented. Blood pressure was 104/50 mmHg, and pulse was irregularly irregular with rates around 110 beats/minute. He was afebrile and saturating at 98% on room air. His examination was notable for decreased breath sound in the right lung base with dullness to percussion and bilateral 1+ pitting lower leg edema. Initial labs showed only a slightly elevated brain natriuretic peptide (BNP) (Table 1). An electrocardiogram (ECG) showed atrial fibrillation with a rapid ventricular rate of 108 beats per minute and right axis deviation (Figure 1).

**Table 1:** Initial labs in the emergency department

Initial Labs	Value	Reference Range
Troponin	<0.03 ng/mL	<=0.06 ng/mL
Brain natriuretic peptide (BNP)	143 pg/mL	0 - 100 pg/mL
Thyroid-stimulating hormone	2.880 µIU/mL	0.450 - 5.330 µIU/mL
Albumin	3.3 g/dL	3.5 - 5.7 g/dL
Aspartate transaminase	4 IU/L	13 - 39 IU/L
Alanine transaminase	4 IU/L	7 - 52 IU/L
Creatinine	0.50 mg/dL	0.60 - 1.30 mg/dL

**Figure 2:**
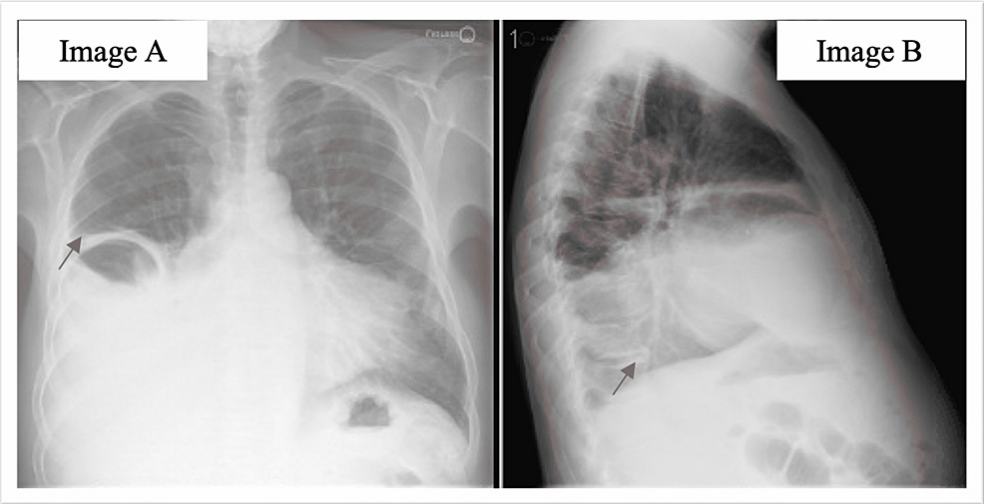
X-ray chest with red arrows in panels A and B showing large right pleural effusion with concerns for consolidation vs. atelectasis secondary to the effusion of the right lung base

His chest X-ray showed a large right pleural effusion with concerns for consolidation versus atelectasis secondary to the effusion of the right lung base (Figure 2).

**Figure 3:**
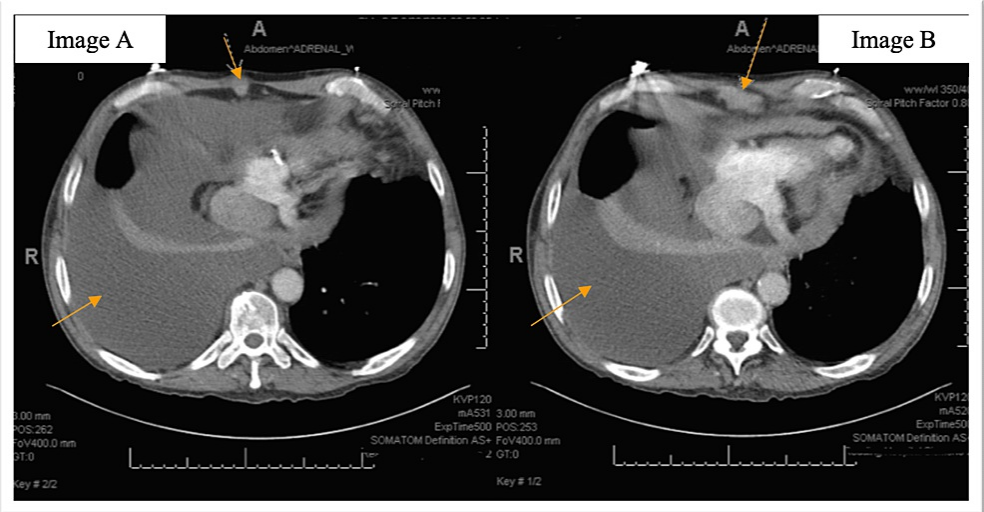
Orange arrows in panels A and B pointing toward an anteroinferior pericardial mass, with concerns for neoplasm and a large right pleural effusion. Yellow arrows in Figures A and B showing right pleural effusion.

He was started on a diuresis regimen, and rate control was achieved with atrioventricular (AV) nodal blocking agents. To further characterize the effusion, he underwent computed tomography (CT) scan of the chest with contrast, which showed moderate to large right pleural effusion with clear lung fields. An anteroinferior pericardial mass was also noted, with concerns for neoplasm (Figure 3).

**Figure 4:**
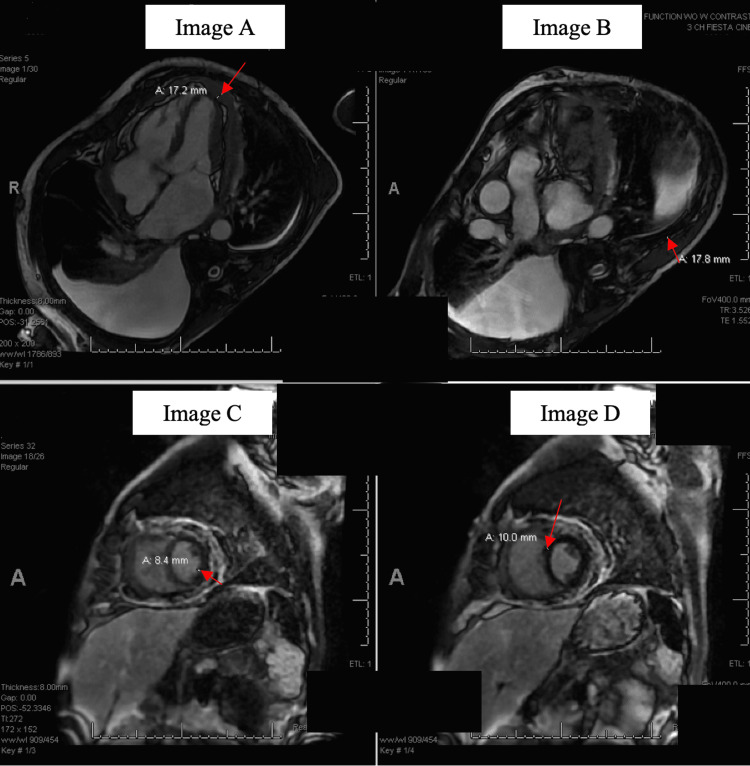
Red arrows in panels A, B, C, and D showing diffusely thickened, heterogeneously enhancing, and irregular pericardium measuring maximally 17.8 mm (in Figure B).

The patient underwent thoracocentesis with 2800 ml of fluid recovery for right-sided pleural effusion. Analysis of the fluid revealed an exudative effusion as per Light's criteria (pleural protein/serum protein ratio of 0.51, pleural lactate dehydrogenase (LDH)/serum LDH ratio of 0.58) with cytology negative for malignancy. No bacterial growth was seen on Day 2 of pleural culture, and a normal procalcitonin of 0.31 ng/ml (normal range: < 0.51 ng/ml) ruled out an infection. Because of our concerns about heart failure exacerbation, we performed transthoracic echocardiography, which showed an ejection fraction of 60% with septal bounce, mild mitral regurgitation, severe tricuspid regurgitation, estimated pulmonary pressure of 35 mmHg, and small pericardial effusion. There was dilatation of the inferior vena cava with paradoxical interventricular septal movement during early diastole with reversal of the relationship between lateral and medial mitral annular velocities (septal E prime was 14, and his lateral wall E prime was 8.8) known as annulus reversus. These findings were concerning for the possibility of constrictive pericarditis. Hence, to establish a definite diagnosis, he underwent right and left heart catheterization for hemodynamic assessment, particularly to assess constrictive physiology. Mean right atrial pressure was 13 mmHg with a prominent y descent. The right ventricular pressure was 35/13 mm Hg with a dip in plateau configuration. Pulmonary artery pressure was 36/19 mmHg, with a mean of 24 mmHg, mean wedge pressure was 16 mmHg, and left ventricular end-diastolic pressure was 17 mmHg. There was an equilibration of diastolic pressures across all cardiac chambers. These hemodynamic findings were consistent with constrictive pericarditis. However, confusion arose on simultaneous tracking of the right ventricle and left ventricle pressure, which showed concordant respiratory variation, more consistent with restrictive physiology. Because of this difficulty differentiating constrictive pericarditis from restrictive cardiomyopathy, cardiac magnetic resonance imaging (MRI) was ordered, which showed diffusely thickened, heterogeneously enhancing, and irregular pericardium measuring maximally 17 mm. A trace amount of loculated pericardial effusion was present between the thickened visceral and parietal pleura. The real-time MRI showed a limited diastolic filling with positive "septal bounce" (Figure 4). All of these findings were supportive of a constrictive pericarditis picture.

**Figure 5:**
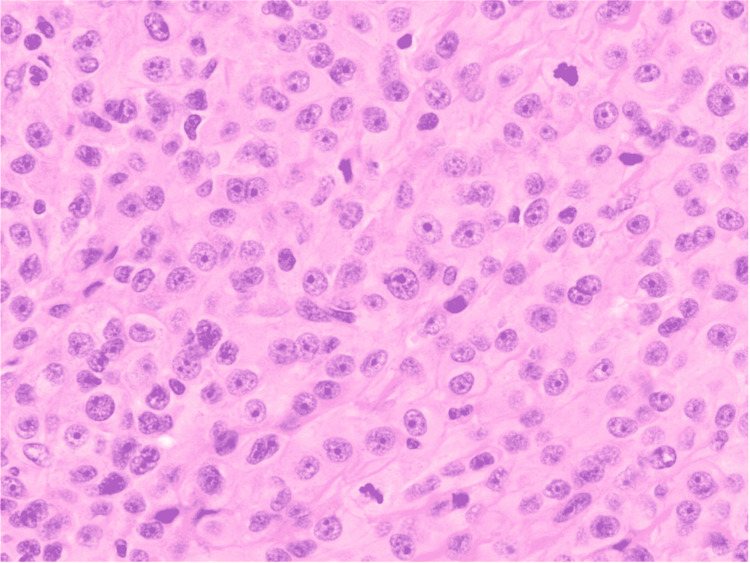
Epithelioid mesothelioma with pleomorphic cells and mitotic activity (hematoxylin-eosin, original magnification 400 X) Pathology: Microscopic sections of the pericardial mass reveal epithelioid and sarcomatoid mesothelioma. A panel of immunoperoxidase stains shows positive staining for pan keratin, vimentin, CK7, and calretinin. The malignant cells show negative staining for LCA, SOX10, MART-1, WT1, CK20, monoclonal CEA, STAT6, TTF-1, and BerEP4. This immunoprofile is diagnostic of malignant mesothelioma. The tumor shows abundant mitotic activity, cellular pleomorphism, and tumor necrosis.

We planned pericardiectomy for constrictive pericarditis and a maze procedure for atrial fibrillation. The surgeon noted thickened pericardium during the procedure, particularly on the right side, with dense adherence to the heart, limiting the surgery. Frozen section analysis showed malignant neoplasm. Histology showed malignant pericardial mesothelioma mixed epithelioid and sarcomatoid type (Figure 5). Panels of immunoperoxidase stains showed positive staining for pan keratin, vimentin, CK7, and calretinin. Negative staining was noted for leukocyte common antigen (LCA), sry-related HMG-Box gene 10 (SOX-10), melanoma antigen recognized by T-cells 1 (MART1), Wilms' tumor suppressor gene1(WT-1), cytokeratin 20 (CK20), monoclonal carcinoembryonic antigen (CEA), signal transducer and activator of transcription 6 (STAT6), thyroid transcription factor 1 (TTF-1), and epithelial cell adhesion molecule/EPCAM (BerEP4). This immunoprofile was most consistent with malignant mesothelioma. He was started on chemotherapy with nivolumab; however, he opted for hospice due to his worsening functional status. He passed away four months after diagnosis.

## Discussion

This case report illustrates the presentation of pericardial mesothelioma, a rare tumor with estimated prevalence based on an extensive review of 500,000 autopsy cases of around <0.0022% [3]. It is more common in men than women, with a 2:1 proportion [4]. The risk factors are asbestos exposure and radiation therapy [5]. Pericardial mesothelioma can be localized or a diffused mass. There are three histological types: spindle cell, epithelial cell, and mixed [6]. The diagnosis can be missed because of nonspecific symptoms; hence, it is often diagnosed late with features of cardiac tamponade, constrictive pericarditis, or heart failure [7]. On literature search, 75% of all pericardial mesothelioma cases are diagnosed on postmortem [8]. We can reduce this by doing pericardial tissue biopsy in patients with recurrent cardiac tamponade and treatment-resistant pericarditis [9]. In our case, the history of pericardial window done for recurrent effusion may have been the initial presentation of pericardial mesothelioma. Echocardiography remains the initial investigative tool, and Kong et al. reported in a retrospective analysis that the findings are usually nonspecific with pericardial effusion (85.9%), pericardial masses (36.4%), and thickening (17.3%), respectively [4]. In our case, the echo showed thickened pericardium dilatation of the inferior vena cava, paradoxical interventricular septal movement during early diastole, and reversed the relationship between lateral and medial mitral annular velocities, which is suggestive of constrictive physiology. On cardiac catheterization, we found prominent y descent and equilibration of diastolic pressures across all cardiac chambers, suggesting constrictive pericarditis; however simultaneous tracking of the right ventricle and left ventricle pressure showed concordant respiratory variation, which is found in restrictive cardiomyopathy, confounding our picture.

Hence, we performed a cardiac MRI, which showed thicked pericardium with a limited diastolic filling with positive "septal bounce," suggesting constrictive pericarditis. The diagnostic value of pericardiocentesis is limited, with the reported positive rate of pericardiocentesis fluid analysis for malignancy has been reported at around 20.9% [4]. Hence, tissue biopsy with histopathological and immunohistochemistry is necessary to establish a diagnosis [7]. There has been no established consensus on treatment to date [10]. The tumor responds poorly to radiotherapy [10]. Combination chemotherapy may reduce the tumor burden. However, survival depends on the possibility of complete tumor resection, which is rarely possible [1]. Hence, despite the optimized therapy, prognosis remains poor, with survival around six months after diagnosis [1,9].

## Conclusions

Pericardial mesothelioma is often diagnosed late because of nonspecific symptoms/signs. The pericardial fluid analysis is positive only in about 20.9% of cases. Hence, a tissue biopsy is needed to establish the diagnosis. There has been no established consensus on treatment to date. The response to therapy also seems to be poor and results only in palliation of symptoms with a median survival of six months from diagnosis despite optimum medical management.
